# Modeling the dynamics of contractual relations

**DOI:** 10.3389/frai.2023.1042319

**Published:** 2023-04-11

**Authors:** Cristine Griffo, Lauro César Araujo, Samuel Meira Brasil, Mamede-Lima Marques, Giancarlo Guizzardi, João Paulo A. Almeida

**Affiliations:** ^1^Faculty of Computer Science, Research Centre for Knowledge and Data (KRDB), Free University of Bozen-Bolzano, Bolzano, Italy; ^2^Center for Data Processing of the Federal Senate, Prodasen, Brazilian Government, Brasília, Brazil; ^3^Court of Justice of the State of Espírito Santo, Vitoria, Brazil; ^4^Modal Institute for Science Technology and Innovation, Brasília, Brazil; ^5^Faculty of Electrical Engineering, Mathematics and Computer Science, University of Twente, Enschede, Netherlands; ^6^Department of Computer Science, Ontology and Conceptual Modeling Research Group (NEMO), Federal University of Espírito Santo, Vitoria, Brazil

**Keywords:** UFO, UFO-L, legal power, legal relation, contract dynamics, contractual changes, unilateral contractual changes

## Abstract

Contracts usually have clauses that enable contracted parties to adjust their contractual positions in time, e.g., to relieve another party from duty or to grant new permission. This is especially important in long-running service relations, which require contracts to be adjusted to accommodate new or unforeseen circumstances. Despite that, the representation of dynamic aspects of contractual relations has not been given enough attention in the literature. In this study, we address this gap by employing the notions of legal power and legal subjection. We propose an ontological analysis of unilateral contractual changes based on a well-founded legal core ontology that adopts a relational perspective for legal positions. We present a case study to show the benefits of representing different types of contractual changes and how these changes can impact contractual dynamics. The case study is based on recent changes to WhatsApp terms of service.

## 1. Introduction

Services have an important role in advanced economies. It is not surprising that deontic aspects of service relations are captured in legally-binding contracts. Contracts establish various legal positions of signing parties, regulating their behavior in the scope of the legal relation.

Over the years, a number of modeling techniques and formalisms have been devoted to the representation and rigorous analysis of contracts. Despite that, there is a gap in the representation of dynamic aspects of contractual relations. Techniques often assume a particular snapshot of a contractual relation, and focus on the deontic positions at a particular point of time not capturing contract changes, such as those promoted by service providers with new versions of service agreements or the enactment of clauses that regulate contract amendment. The lack of support for contractual dynamics is detrimental to real-world applications, which require knowledge representation to faithfully represent the relevant complexities of the targeted real-world phenomena.

In this study, we address the representation of the dynamics of contracts through the notion of legal power and its correlative notion of legal subjection (Griffo et al., 2022). A legal agent is said to hold power toward another party when it is capable of action that legally alters (creates, changes, removes) the legal positions of that party (Alexy, 2009). The party liable to change in legal positions is said to be *subjected to* the power holder in that *power–subjection relation*.

The representation of the contractual dynamics proposed in this paper is based on the reification of legal relations according to the Unified Foundational Ontology (UFO), and the legal core ontology (UFO-L) developed to address legal phenomena in general (Griffo et al., 2016, 2019), and on the Service Core Ontology (SCO) to address legal aspects of service contracts (Griffo et al., 2019). A Legal Service Agreement is considered in this case a complex legal relation formed by simpler legal relations of various types (Right–Duty, No-Right–Permission, Disability–Immunity, and Power–Subjection). These relations exist by virtue of the contract that is agreed upon by contracting parties, which may be changed throughout its lifecycle. Each type of relation is associated with a representational pattern (Griffo et al., 2016), leveraging the reuse of a well-founded modeling solution.

The choice for the ontological approach is based on the premise that ontologies allow explicit semantic representation structures of a domain. These representation structures allow the semantics of things and the relations between them to be brought to light. Moreover, considering that *good ontologies* (Guarino et al., 2009) are built on the basis of well-founded ontologies, foundational ontologies, such as DOLCE (Borgo and Masolo, 2009) and Unified Foundational Ontology (UFO) (Guizzardi, 2005), are used to bring consistency and coherence to domain-dependent ontologies, also called domain ontologies. In this regard, several empirical studies have been confirmed the benefits of using ontologies, such as the study of Griffo et al. (2018), which investigated the quality of models built with the UFO-L patterns for legal relations in terms of correct interpretation of contracts, the improving of answering performance, and the perceived clarity in contract clauses; and Verdonck et al. (2019), who conducted an empirical study that explored the differences between the traditional conceptual modeling (TCM) technique and an ontology-driven conceptual modeling (ODCM) technique. Moreover, ontologies have been used in successful applications in different sectors (Guizzardi et al., 2015).

To verify the application of the UFO-L power–subjection pattern, a case study involving the changes in WhatsApp's terms of services that occurred in 2021 was conducted. One of the main aims of this study is to show that the terms of service are not drafted in such a way as to make explicit the service customer's subjection positions and that the use of the UFO-L power–subjection pattern can make explicit the legal positions involved, the types of changes, and the mechanism of change, inclusion, and extinction of legal relations in the scope of service provisioning.

In summary, this study considers the existing contributions in the literature on the dynamics of contractual relations. However, it focuses on a relational ontological approach to explore how to represent contractual dynamics based on an ontological theory of relations. While previous studies on contractual dynamics focused on violations of deontic commands existing in contractual clauses, as seen in *Contract Language (CL)* (Prisacariu and Schneider, 2012), *Business Contract Language (BCL)* (Governatori and Milosevic, 2006), and *Formal Contract Language (FCL)* (Farmer and Hu, 2016), this study emphasizes contractual dynamics representation based on legal relations, in particular, the dynamics of legal relations of power and subjection existing in contractual clauses of unilateral changes, as well as in the representation of the intrinsic aspects of each role performed by the contracted parties and in the possible violations of these intrinsic aspects.

Thus, the main contributions of our study to the problem of contractual dynamics are as follows: (1) an ontological analysis of types of contractual changes; and (2) the representation of the contractual dynamics resulting from unilateral contractual changes.

As we explained in the study of Griffo et al. (2019)^1^, there are some legal positions not covered by deontic-logic-based contractual languages, such as *permission, no-right, power, and submission*, which appear from simple legal documents to complex ones, and thus must be represented in languages or ontologies intended to represent contracts in general. By admitting those positions fully, we address limitations in representations that are solely based on deontic logic and make it possible to account for contractual changes.

This study is further structured as follows: Section 2 presents the foundations we employ, introducing fragments of the reference ontologies UFO, UFO-L, and SCO that are relevant here; Section 3 presents an analysis of WhatsApp's terms of service change in light of the foundations; Section 4 reviews related works, comparing with our approach, and discusses gaps in the representation of the dynamics of contractual relations; finally, Section 5 provides concluding remarks and outlines future study.

## 2. Theoretical framework

In this study, we introduce the ontological representation for two different types of legal procedures of modifications in agreements: *amendment* and *novation*, which are not discussed in our previous studies. At the same time, we apply the pattern proposed for legal power-subjection relations (Griffo et al., 2022) and point out the benefits of applying it. For the sake of space, we restrict the scope of this study, analysis, and representation of *unilateral modification clauses* of service agreements initiated by a *Service Provider*, employing the legal power contained in power–subjection legal relations. We will address in future works other contractual modification types, e.g., contractual modifications arising out of mutual consensus.

### 2.1. Contractual amendment and novation

In contract management, it is relevant to keep a record of amendments in the original contract, i.e., which legal clauses were changed, added, or removed, and consequently the alterations in the legal positions related to them. In addition, it is important to create some mechanism for tracking new contracts which are – somehow – linked with previous contracts. For instance, the change of contracting parties can generate a new contract. Keeping the link between contracts with this type of change can be interesting for tax purposes or internal managing the contracts themselves.

A *unilateral contractual amendment* is a legal procedure that adds, removes, or alters parts of the original contract. It replaces the specified portion of the original contract and keeps the continuity of the overall legal relation. On the contrary, a *contractual novation* is a mode of discharging contracts with the consent of the parties. The effect of novation is a new contract with a new legal relation and the discharge of the old contract (Meena, 2008).

Generally, in *adhesion* service contracts such as Internet provisioning, app subscription, telephony services, etc., a clause is included to give the service provider the means to promote unilateral contract modification. This clause should be understood as establishing a legal relation of power and subjection, where the power holder is the service provider and the subjection holder is the customer. Therefore, the bargaining power is asymmetric in such types of contracts.

U.S. Courts have often interpreted adhesion contracts restrictively, applying protection theories to the weaker contractual party, for example, the Reasonable Expectations Doctrine (Fett, 1992) as a basis for nullifying contractual clauses or the entire contract. In addition, the European Parliament and the Council of the European Union have adopted Directive 2019/770 to rule the digital services and establish requirements concerning digital service contracts. Regarding contractual modifications, Article 19 of the Directive establishes some conditions to be observed in case of contractual modifications, among them, ‘* (b) (...) modification is made without additional cost to the consumer;'*. “Cost to the consumer” also includes in this case the value of intangible assets that are owned by the consumer, such as his/her information. For example, a service provider violates Article 19 item(b) when adding a contractual clause in which the consumer shall permit the sharing of his/her information with the service provider's partner companies. This is because, with this modification, the service provider is burdening the consumer.

With regard to contractual modification procedures, some papers published in law journals and conferences have discussed the problem of amendment clauses, such as the study of Horton (2009) that exposes such clauses in adhesion contracts and points out the existence of a “pernicious feedback loop”, in which each judicial decision annuls a contractual amendment, contract drafters respond by amending the terms of service again, rendering court rulings ineffective and thus emptying the judicial decisions.

Hence, one question that arises is what the boundary between *amendment* and *novation* is, and how clearcut it is. This issue is relevant due to the fact that the lack of clarity and the contractual imprecision increase the litigation level. In Brazil, for instance, contractual imprecision is considered one of the four most common reasons for consumer litigation (CNJ, 2021).

### 2.2. Ontologies and ontological languages

We ground our research in a foundational ontology called *Unified Foundational Ontology (UFO)* (Guizzardi, 2005; Guizzardi et al., 2018) as well as UFO-L, a core ontology of legal aspects (Griffo et al., 2017) based on UFO and the theory of constitutional rights proposed by jurist Alexy (2009). From these ontologies, the ontology of service contracts (called *Service Contract Ontology* (SCO)) was built (Griffo et al., 2019).

Unified Foundational Ontology represents the Aristotelian square of *Individuals* and *Universals* where *Individuals* are instances of *Universals*. In UFO, relations are reified (i.e., treated as object-like entities) by means of *relators*. The conceptual modeling technique to reify entities of a certain domain brings the benefit of the clarity of these entities that, otherwise, could be implicit in the domain of discourse. In particular, the reification is performed on those entities that carry true content for the propositions existing in the domain to be represented. These entities are called *truth-makers* (Guarino et al., 2018).

The UFO-L follows this proposition by reifying legal relations. Thus, a legal relation is reified by means of a *legal relator* (Griffo, 2018), making it possible to represent the life cycle of the contractual relations as *endurants*, i.e., the identity (essence) of these entities is not changed over time, even if its accidental properties are changed. For example, the service contract between Mary and WhatsApp remains the same contract even if Mary changes phones or there are unilateral or bilateral non-essential changes to the contract.

In UFO-L (Griffo, 2018; Griffo et al., 2019), there is a set of types of legal relators as follows: (1) *Simple Legal Relator*: *Right–Duty relators, NoRight–Permission relators, Power–Subjection relators* and their subtypes; and (2) *Complex Legal Relator*: Unprotected Liberty relators and Protected Liberty relators. Simple legal relators are composed of legal positions *(Right, Duty, NoRight to an Omission, Permission to Act, Power, and Subjection*, among others), and complex legal relators are composed of other legal relators. For instance, liberty relators are complex legal relators composed of a set of permission relators (Permission to Act–NoRight to an Omission legal relation and Permission to Omit–NoRight to an Action). The liberty to contract is a composition of permission to contract and permission to not contract (and its correlated positions).

The reason why the terminology proposed by Hohfeld for liberty was not used is that *liberty* means a privilege to act **or** a privilege not to act (‘*A “liberty” considered as a legal relation – or “right” in the loose and generic sense of that term – must mean, if it have any definite content at all, precisely the same thing as privilege*') (Hohfeld, 1913). In this sense, UFO-L approaches Alexy's system of basic legal positions, which defines liberty as a composition of permissions to act **and** not to act.

Regarding ontologies of services, UFO-S is a reference ontology intended to assist humans in meaningful negotiation and shared understanding. It is grounded in UFO, from which it reuses foundational notions of objects, types, object properties, object relations, reified relational complexes (relators), and events/processes, and further social concepts that specialize the more general notions and account for social reality. In UFO-S, service relations are specializations of social relations, which are, in turn, material relations. There are three basic phases of the service life cycle represented in UFO-S, namely, (i) service offer (when a service is presented and made available to a target customer community), (ii) service negotiation (when providers and customers negotiate in order to establish an agreement), and (iii) service delivery (when actions are performed to fulfill a service agreement).

**Figure 1:**
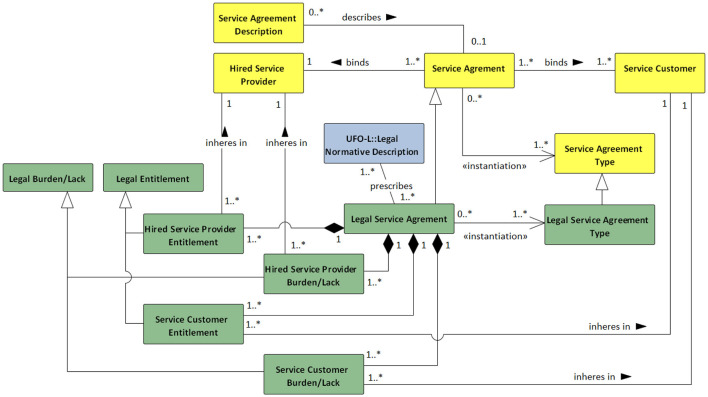
SCO: Legal service agreement (fragment).

Service Contract Ontology (SCO) (Figure 1) is an ontology grounded on UFO, UFO-S, and UFO-L with the purpose to represent contracts. A contract is a *Legal Service Agreement*, which is a subtype of *Service Agreement* and an instantiation of the *Legal Service Agreement Type*. Therefore, there are *Service Agreements* that go beyond the social dimension and reach the legal dimension. These are the subtypes defined as *Legal Service Agreement*. These ones are ruled by *Legal Normative Descriptions* (e.g., directives, acts). In addition, *Legal Service Agreements* are composed of *legal burdens/lacks* and *legal entitlement*, legal aspects inherent in the roles played by the bound parties (*Hired Service Provider* and *Service Customer*) (Nardi et al., 2015, 2016b).

A *Service Agreement Description* describes a service agreement, exposing the main elements of an agreement, such as the contractual parties, the object of the agreement, conditions, penalties, and all sorts of relations that bind a *Hired Service Provider* and a *Service Customer* (the yellow portion in Figure 1) (Nardi et al., 2015, 2016b). Although a *Service Agreement Description* contains the elements of an agreement, it is not the agreement itself. This is because there is a difference between the ontological nature of the *Service Agreement* and *Service Agreement Description*. The first one has an ontological nature of the *relation* and the second one has an ontological nature of *object*; a service agreement Description is a text while the Service Agreement is the relation itself described in the Service Agreement Description. Furthermore, there are types of service agreements that have legal effects, such as terms of services between a provider of communication apps and customers. In these type of service agreements, called *Legal Service Agreement*, the legal relation is *prescribed* by legal norms established at an upper level (meta-norms), and the parties, under meta-norms, can stipulate a set of particular norms, which make law between the parties, i.e., the contract is a law *in concretum*.

These agreements must necessarily be written considering requirements prescribed by law. In Figure 1, a *Legal Service Agreement* (in green) specializes *Service Agreement* and instantiates a *Legal Service Agreement Type*. A Legal Service Agreement (LSA-relation) is a bundle of legal relations prescribed by a Legal Normative Description (e.g., the document *Terms of Services* drafted by WhatsApp is a Legal Normative Description but the content of this document is a bundle of legal relations). In addition, an LSA-relation legally binds *Hired Service Provider* and *Service Customer*.

**Figure 2:**
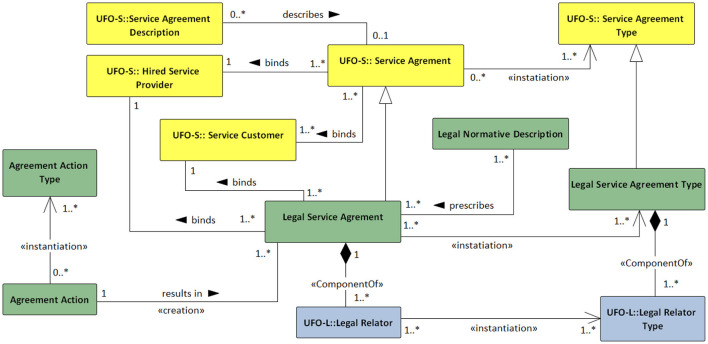
SCO: Legal service agreement (fragment).

A Legal Service Agreement is created by an *event* called *Agreement Action*. Agreement actions are, for instance, the signature of the parties in a contract, the verbal accordance, and the *silence* when circumstances or usage authorize it, and an express statement of intent is not required to establish an agreement. Some *Service Agreements* are not legally binding, for example, if John commits to help Mary fix her computer out of friendship, in this case, there is no legal relation binding them nor a *Legal Normative Description* describing the relation. On the contrary, there are service agreements that have the power of law behind them, and therefore, they have a normative description enforcing the law on each term of the agreement (Figure 2).

In SCO (Figure 1), legal positions are grouped in two types of legal positions as follows: (1) *Legal Entitlement*, which is specialized in *Permission, Right, Immunity, and Power*; and 2) *Legal Burden/Lack*, which is specialized in *Duty, NoRight, Disability, and Subjection* (Griffo et al., 2019). A Legal Service Agreement is composed of *Hired Service Provider Entitlement, Hired Service Provider Burden/Lack, Service Customer Entitlement*, and *Service Customer Burden/Lack*, as shown in Figure 1 (the green part).

At the instance level, each instance of the *Legal Service Agreement* binds both instances of *Service Customer* and *Hired Service Provider*. In addition, an instance of the *Legal Service Agreement* is composed of instances of legal positions. By modeling in this way, it is possible to clarify the various existing aspects of a legal relation (Griffo et al., 2019).

An interesting issue of modeling legal service agreements is the representation of legal service agreement subtypes (see 3.3, Figure 6, the portion in green). Each Service Provider drafts its own legal service agreements according to its preferences. This means that a subtype of LSA-relation between a Service Provider and its Services Customers specializes in Legal Service Agreement. For example, WhatsApp's Legal Service Agreement and Amazon's Legal Service Agreement are subtypes of Legal Service Agreement. In addition, WhatsApp's Legal Service Agreement can have versions. For instance, a WhatsApp's LSA-relation dated 28 January 2020 did not establish permission for WhatsApp to share the service customer's information to third parties, but in WhatsApp's LSA-relation dated 4 January 2021, this legal position is present.

Another issue is the difference in modeling *novations* and *amendments*. As distinguished in Section 2.1, *amendments* add, change, or exclude clauses from the original contract without destroying the legal relation between the contracting parties. In contrast, *novations* alter the original contract, replacing the original legal relation with a new one. Hence, in amendments, it is necessary to model *phases* of a Legal Service Agreement (LSA-relation). The same agreement will go through these phases during its lifetime. On the contrary, in novations, it is necessary to represent different *kinds* of Legal Service Agreements (i.e., different Legal Service Agreement Types.) Hence, we do not speak of the same agreement but different agreements, related historically.

### 2.3. Legal power-subjection relations

One of the significant theoretical references for this study is the theory of fundamental legal positions proposed by Hohfeld (1913). Hohfeld presented a taxonomy composed of eight ‘fundamental‘ concepts relying on notions of two types of relations: opposition and correlation. One of these legal positions is *power*, which also takes other names, such as legal power, authority, competence, and legal capacity, among others. In a previous article, we discussed the opposition relations in the study of Hohfeld and presented some contributions related to *competence*. In our scheme, we consider not only the competence to change a legal position–with the correlative *liability* but also the impossibility of changing it, which is the disability, with the correlative *immunity*.

**Figure 3:**
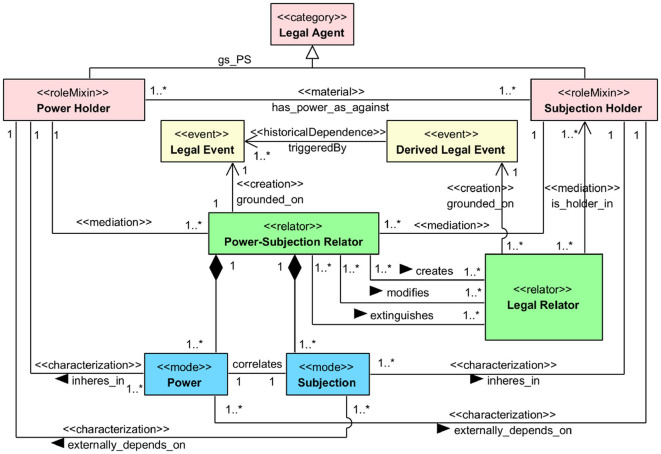
UFO-L pattern: Legal power-subjection relator (Griffo et al., 2022).

In this present article, the attention falls on the correlation relations, in particular, on the legal power-subjection relations in service contracts. The ontological analysis of that type of legal relation as well as a conceptual modeling pattern was previously published in the study of Griffo et al. (2022). Figure 3 shows the legal power-subjection relation pattern based on Alexy's system of basic positions and Hohfeld's taxonomy. In UFO-L, legal powers are defined as special types of legal aspects inherent in roles played by legal agents. Their exercise occurs by means of institutional acts, (Searle, 1995; Alexy, 2009) whose types (and consequent situations) are explicitly prescribed in *legal normative descriptions*. In other words, legal powers are ‘artificial capabilities' granted by means of institutional acts (Directives for the European Parliament, for example), with the purpose of creating, altering, or extinguishing legal relations in which the opposite legal agent is a part. There are two subtypes of legal Power-Subjection relations as follows: if simple, they are composed of correlative power-subjection pairs (inhering in opposing roles played by agents); if complex, they are composed of legal relations.

Legal aspects are linked to *legal roles* played by *legal agents* by means of a type of a relation of *inherence* (a type of *existential dependence* represented here by the relation of *characterization*) and connected to the other *legal roles*, participant of the same relation, by means of a relation of external dependence (*externally depends on*). Thus, legal power is an aspect that externally depends on Subjection Holder, and legal subjection is an aspect that externally depends on Power Holder. With these associations, although these aspects are inherent in the mentioned legal roles, they occur in relational pairs.

## 3. Case study: WhatsApp's agreement changing

The analysis of WhatsApp terms of service is the second study carried out with service contracts by the authors. The first study was Amazon's terms of services with the scope of extending the concrete syntax of ArchiMate (Griffo et al., 2017). In the second study, the scope is different. Here, the purpose was to present how the representations of two types of contractual modifications—amendment and novation—are built using UFO/UFO-L. These representations can be used as support for contract management and allow making inferences about consumers who adhered to the modifications and the intrinsic aspects of each legal relation, just to name a few applications. Indeed, these terms of services provide an interesting field of study by raising cardinal questions, such as, what legal positions are being modified?, what is the chain of events that occur with a contractual modification? Or what exists for a contractual modification to occur?

Regarding contractual writing, clauses of power and subjection in real terms of service (TS) are not drafted in such a way as to make explicit the service customer's subjection positions. In this case study, we present an ontological representation approach that makes them clearer. We advocate that the use of the UFO-L power-subjection relator pattern to represent this type of legal relation makes clearer the legal positions involved, as well as the mechanism of changes of legal relations derived from this legal relation. To defend this claim, we conducted a case study on the WhatsApp terms of services, represented the power-subjection relations found in the contractual clauses, applying the UFO-L power-subjection relator pattern.

WhatsApp is a platform for instant messaging and voice calling apps for smartphones. It provides ways for its Service Customers to communicate with other WhatsApp Service Customers through messages, voice and video calls, sending images, documents, and videos, showing your status, and sharing your location with others. In 2014, WhatsApp was purchased by (formerly) Facebook Companies and after that, it has also included in its portfolio services to send and receive money to or from other service customers across WhatsApp's platform; also, services to connect with services of WhatsApp's partners, other service providers, and affiliated companies.^2^

The methods, strategies, and tools used to conduct this study can be applied to other contracts and terms of services, including those typical of FAANG platforms (Facebook/Meta, Apple, Amazon, Netflix, and Google/Alphabet) or yet non-digital platforms. The representation built is not conditioned to the technology or service or particular object of the contract. We have chosen WhatsApp's Terms of Service (WTS) as a case study due of the presence of clauses with legal power-subjection relations, beyond the fact that WTS is a well-known service as well as its recent updates in terms of service.

### 3.1. Dynamics of WhatsApp terms of services

The WhatsApp terms of services, presented in Table 1, have a clause that allows unilateral modification of contractual clauses by WhatsApp. When WhatsApp exercises the legal position of power implied by that clause, it generates another legal document that overlaps the previous one. In this new legal document, there may be new clauses, and therefore new legal relations, changes to clauses existing in the previous agreement, or the removal of clauses, and consequently, the termination of legal relations. The question is: *what is the legal nature of this modification?* Is it a contractual amendment or a contractual novation?

**Table 1:** Terms of service published by WhatsApp.

**WhatsApp terms of service**	
**Non-European**	**European**
04 January 2021^1^	04 January 2021^2^
28 January 2020^3^	24 April 2018^4^
25 August 2016	25 August 2016
7 July 2012	7 July 2012

1 EEA-Revision-Feb 2021.

2 EEA-Revision-Feb 2021.

3 current non-European version.

4 current European version.

**Figure 4:**
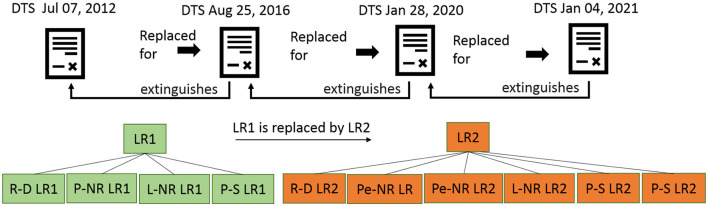
WhatsApp terms of services – novation.

If the legal nature of the modifications in the WhatsApp terms is *novation*, then for each new document published, there is a new legal relation (LR) between WhatsApp (SP) and a service customer (SC), as shown in Figure 4. In novation, for each new version of the document, a new complex legal relation (LR) is created. Some of its parts may be similar to those in the previous complex legal relation; some others are dropped, added, or significantly changed.

**Figure 5:**
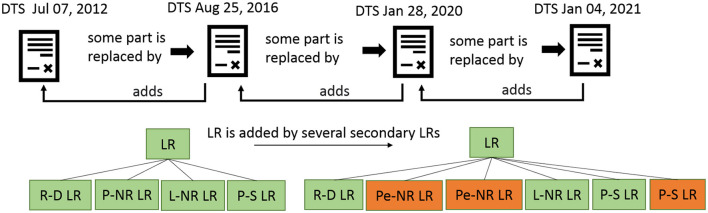
WhatsApp terms of services – amendment.

On the contrary, if the legal nature of the modifications in the WhatsApp terms is *mendment*, the complex legal relation between WhatsApp and a service customer is the same, changing only parts of this complex legal relation. For instance, Figure 5 shows a term of service TS1 that defines and prescribes a complex legal relation LR1. This legal relation is composed of other legal relations: right–duty relation (R–D LR), permission–no-right relation (Pe-NR LR), unprotected liberty–no-right relation (L–NR LR), and power–subjection relation (P–S LR). If TS1 is amended by TS2, TS2 includes other clauses in TS1 (in orange). In amendments, the dynamics of Legal Service Agreements can be understood as the existence of several valid documents for the same legal relation, which has its parts modified.

We have identified some aspects of the dynamics of WhatsApp's terms of services, which are as follows:

1. WhatsApp has the power to change the terms of services. The Service Customer, by consenting to the change, subjects him/herself to the new WTS.
2. In general, contracts have an essential part, with clauses that cannot be changed without denaturalizing the object of the legal relation; and an accidental part, with clauses that can be changed without denaturalizing the object of the legal relation. It is not explicit in WTS which part is essential and which part is accidental.
3. At a given time, there can only be one WTS. The *document* of the WTS (DWTS) is not to be confused with the bundle of legal relations created, defined, and prescribed by this document.
4. In a DWTS chain, the most recent DWTS revokes the previous DWTS in what is contrary.

### 3.2. Instruments and strategies

Table 1 shows various dates involving changes to the “WhatsApp terms of service” (WTS). There are two types of WTS by region: a non-European WTS version and a European WTS version. For the first one, there are four versions dated: 4 January 2021; 28 January 2020 (after the purchase of WhatsApp by Facebook Co.); 25 August 2016; and 7 July 2012. From this list of WTS versions, we selected the last and penultimate versions of the non-European WTS versions to conduct the case study (it is noteworthy that we did not analyze the bind arbitration clause existing in WTS, specifically for Canada and the United States).

Regarding ontological terms used in this study, we applied some terms coined in UFO-S (Nardi et al., 2016a), such as Service Provider and Service Customer, which we have also used in previous study on service contracts (Griffo et al., 2019). Thus, regarding the contractual parts, in the version of DWTS concerning the non-European region, the Service Provider is WhatsApp LLC. In turn, in the European region, the Service Provider is WhatsApp Ireland Limited. In both regions (European and non-European regions), WhatsApp users (or clients) are the Service Customers.^3^

We conducted this case study taking the following steps:

1. Identification of the WhatsApp Terms of Service (Table 1).
2. Selection of changed clauses in WTS (latest versions of 2020 and 2021) (Tables 2, 4).
3. Identification of the verbs or verbal expressions in the selected clauses (Tables 2, 4) in order to identify legal relation patterns.
4. Classification of the legal relations in the selected clauses of (Tables 2, 4) based on UFO-L concepts.
5. Identification of the existing legal positions in every classified legal relation according to UFO-L patterns (Tables 3, 5).
6. Modeling power-subjection relations applying UFO-L patterns (Subsection 3.3).

**Table 2:** Some clauses of non-European version 04.01.2021.

**ID**	**Clauses**
1	We provide ways for you to communicate (...), sending images and video, showing your status, and sharing your location with others when you choose.
2	We analyze how you make use of WhatsApp, in order to improve our Services, including helping businesses who use WhatsApp measure the effectiveness and distribution of their services and messages.
	WhatsApp uses the information it has and also works with partners, service providers, and affiliated companies to do this.
6	WhatsApp receives information from, and shares information with, the Facebook Companies as described in WhatsApp's Privacy Policy, (...) including to provide integration which enable you to connect your WhatsApp experience with other Facebook Company Products; to ensure security, safety, and integrity across the Facebook Company Products; and to improve your ads and products experience across the Facebook Company Products.
8	You must register for our Services using accurate information, provide your current mobile phone number (...)
36	Any amendment to or waiver proposed by you of our Terms requires our express consent.
37	We may amend or update these Terms. We will provide you notice of material amendments to our Terms, **as appropriate**, and update the “Last modified” date at the top of our Terms. Your continued use of our Services confirms your acceptance of our Terms, as amended. We hope you will continue using our Services, but if you do not agree to our Terms, as amended, you must stop using our Services by deleting your account.

**Table 3:** Classification of clauses from Table 2.

**ID**	**Holder**	**Legal position**	**Legal relator**
1	Duty Holder (SP)	Duty to act	RDA−LR
	Right Holder (SC)	Right to an Action	
2	Permission Holder (SP)	Permission to act	NROPA−LR
	NoRight Holder (SC)	NoRight to an Omission	
	Permission Holder (SP)	Permission to act	NROPA−LR
	NoRight Holder (SC)	NoRight to an Omission	
6	Permission Holder (SP)	Permission to act	NROPA−LR
	NoRight Holder (SC)	NoRight to an Omission	
	Permission Holer (SP)	Permission to act	NROPA−LR
	NoRight Holder (SC)	NoRight to an Omission	
8	Duty Holder (SC)	Duty to act	RDA−LR
	Right Holder (SP)	Right to an Action	
37	Power Holder (SP)	Power	PS−LR
	Subjection Holder (SC)	Subjection	
	Liberty Holder (SP)	Liberty	L−LR
	NoRight Holder (SC)	NoRight to an Omission, NoRight to an Action	
	Duty Holder (SP)	Duty to act	RDA−LR
	Right Holder (SC)	Right to an Action	
	NoRight Holder (SC)	NoRight to an Omission	NROPA−LR
	Permission Holder (SP)	Permission to Act	
	Power Holder (SC)	Power	PS−LR
	Subjection Holder (SP)	Subjection	
	Duty Holder (SC)	Duty to omit	RDO−LR
	Right Holder(SP)	Right to an Omission	
	Duty Holder (SC)	Duty to act	RDA−LR
	Right Holder (SP)	Right to an Action	

SP, Service Provider; SC, Service Customer; RDA−LR, Right−Duty to an action relator; RDO−LR, Right−Duty to an omission relator; NROPA−LR, NoRight to an Omission−Permission to act relator; PS−LR, Power−Subjection relator; L−LR, Liberty relator.

**Table 4:** Some clauses of non-European terms of service version 28.01.2020.

**ID**	**Clauses**
1	WhatsApp LLC (“WhatsApp,” “our,” “we,” or “us”) provides messaging, Internet calling, and other services to users around the world. (...). You agree to our Terms of Service (“Terms”) by installing, accessing, or using our apps, services, features, software, or website (together, “Services”).
3	You must register for our Services using accurate data, provide your current mobile phone number (...).
29	Any amendment to or waiver of our Terms requires our express consent.
30	We may amend or update these Terms. We will provide you notice of material amendments to our Terms, **as appropriate**, and update the “Last modified” date at the top of our Terms. Your continued use of our Services confirms your acceptance of our Terms, as amended. If you do not agree to our Terms, as amended, you must stop using our Services. (...).
31	All of our rights and obligations under our Terms are freely assignable by us to any of our affiliates or in connection with a merger, acquisition, restructuring, or sale of assets, or by operation of law or otherwise, and we may transfer your information to any of our affiliates, successor entities, or new owner.

**Table 5:** Classification of clauses from Table 4.

**ID**	**Holder**	**Legal position**	**Legal relator**
1	Duty Holder (SP)	Duty to act	RDA−LR
	Right Holder (SC)	Right to an Action	
	Power Holder (SC)	Power	PS−LR
	Subjection Holder (SP)	Subjection	
	Liberty Holder (SC)	Liberty	L−LR
	NoRight Holder (SP)	NoRight to an Omission, NoRight to an Action	
3	Duty Holder (SC)	Duty to act	RDA−LR
	Right Holder (SP)	Right to an Action	
	Duty Holder (SC)	Duty to act	RDA−LR
	Right Holder (SP)	Right to an Action	
29	Power Holder (SP)	Power	PS−LR
	Subjection Holder (SC)	Subjection	
	Power Holder (SC)	Power	PS−LR
	Subjection Holder (SP)	Subjection	
30	Power Holder (SP)	Power	PS−LR
	Subjection Holder (SC)	Subjection	
	Liberty Holder (SP)	Liberty	L−LR
	NoRight Holder (SC)	NoRight to an Omission, NoRight to an Action	
	Duty Holder (SP)	Duty to act	RDA−LR
	Right Holder (SC)	Right to an Action	
	Permission Holder (SP)	Permission to Act	
	NoRight Holder (SC)	NoRight to an Omission	NROPA−LR
	Power Holder (SC)	Power	PS−LR
	Subjection Holder (SP)	Subjection	
	Duty Holder (SC)	Duty to omit	RDO−LR
	Right Holder(SP)	Right to an Omission	
31	Power Holder (SP)	Power	PS−LR
	Subjection Holder (SC)	Subjection	
	Liberty Holder (SP)	Liberty	L−LR
	NoRight Holder (SC)	NoRight to an Omission, NoRight to an Action	

SP, Service Provider; SC, Service Customer; RDA−LR, Right−Duty to an Action relator; RDO−LR, Right−Duty to an Omission relator; NROPA−LR, NoRight to an omission−Permission to act relator; PS−LR, Power−Subjection relator; L−LR, iberty relator.

Tables 1, 2, 4 present a fragment of the resulting tables of steps 1 to 3. The WhatsApp's terms of services selected were as follows: WTS non-European 4 January 2021; and WTS non-European 28 January 2020. Some clauses of these terms are shown in Tables 1, 4. In these tables, the ID column is a reference to the full table, which is omitted due to space constraints. For each clause, we highlighted the verbs or expressions, and then, we related each one to a legal relation pattern in UFO-L (Tables 3, 5).

### 3.3. Modeling legal relations

**Figure 6:**
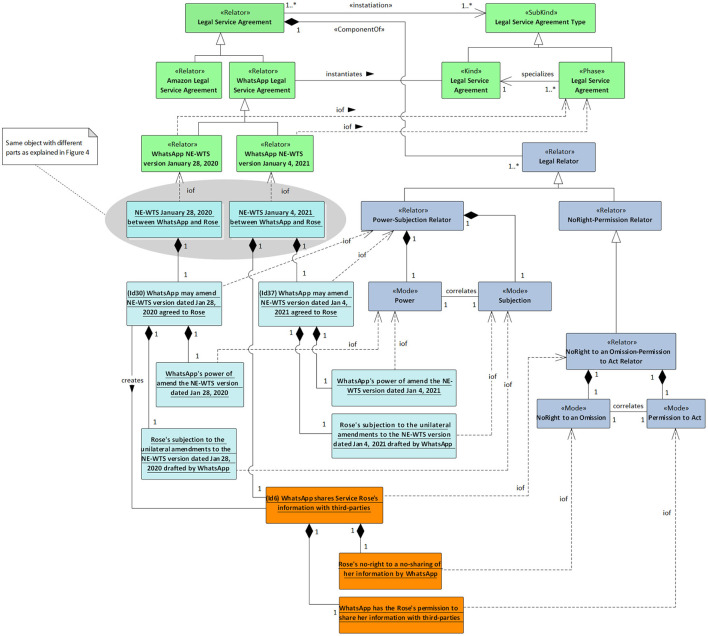
Amendment Scenario in UFO-L.

As we pointed out in Section 2, legal service relations are represented in UFO-L as legal relators. There are two possible representations for the legal relations arising from the modified documents. If these documents have a legal nature of *novation*, the representation is a new legal relation, i.e., a new *Legal Service Agreement*. On the contrary, if these documents have a legal nature of the *amendment*, there is *not* a new legal relation. Instead, the legal relation between WhatsApp and a service customer is the same but with different phases, each one marked by different aspects (e.g., new legal positions) and implemented by the appearance of a different legal document (contractual amendment). In this scenario, there is a new Legal Service Agreement Phase specializing in an existing WhatsApp Legal Service Agreement. Figure 6 shows the modeling of this dynamics, revealing not only the types involved but also some exemplifying instances (the legal relation of WhatsApp with a particular customer, named “Rose” that survives the contractual changes). Notably, we assume further in this analysis that this is a case of amendment, which is consistent with the intent expressed in the usage of the terms ”amend” and ”update” by WhatsApp (Id37) but, more importantly, is the result of unilateral power-subjection clauses included explicitly in the original agreement.

In Tables 2, 4, the relevant power-subjection clauses are Id37 (version 4 January 2021) and Id30 (version 28 January 2020), respectively. We focused on the first power-subjection relation found in these clauses (i.e., “*we may amend or update these Terms.”*). The *Power Holder* is the *Service Provider* WhatsApp, the one who can change the terms unilaterally, generating new legal documents.^4^ By exercising the power established in the Id30 clause, WhatsApp created new clauses (Id 2 and Id6, for example) with new legal positions. Some existing clauses in the version dated 28 January 2020 are repeated in the version dated 4 January 2021 (see Tables 2, 4). In Figure 6, we represented the power–subjection legal relation existing in Id30 and one of the new legal positions created by it (Id6: Permission to share information with third parties).

With respect to contractual changes made by the service customer, they will only be valid with the express consent of the service provider (ID 36, Table 2; and Id 29, Table 4). In this way, the service customer's power to change the WhatsApp Terms of Service is conditioned to WhatsApp's power to want or not the proposed change. In other words, contractual changes made by the service customer will only be valid with WhatsApp's expressed assent. On the contrary, valid contractual changes made by WhatsApp occur not only in the case of the service customer's expressed assent but also with his/her silence added to the continuity of the use of the service.

Figure 6 also reveals that the various versions of the WhatsApp Terms of Service are, in fact, phases of the same agreement, which is a specialization of a more general agreement type. Phases, unlike types, classify their instances contingently. Hence, instances of the phases shown actually denote the same agreement between WhatsApp and Rose that changes qualitatively in time, here gaining new parts (Id6: Permission to share information with third parties; Id30: a Permission–No-Right relation as a consequence of the exercise of power; and Id37: WhatsApp's renewed power to change the contract).

With this modeling, the distinction between things that do not change (the agreement between WhatsApp and Rose) and things that change in a time interval (the non-essential aspects of the agreement between WhatsApp and Rose) remain. Thus, the criterion of identity of an LSA depends on the delimitation of legal relations that are immutable (rigid core that gives identity to the object).

The distinction between *legal agreement kinds* and *legal agreement phases* brings up an important question to be settled: those aspects that characterize the *legal agreement kind* form a rigid core, identifying that which gives identity to the agreement. The lack of delimitation of this rigid core opens the possibility of adopting different identity criteria by the service customer and the service provider, which can generate legal disputes. For example, WhatsApp argues that it has not changed essential parts of the LSA signed with Rose, and therefore, it is the same contract with new clauses (amendment). On the contrary, Rose may argue that WhatsApp changed essential parts as it changed legal positions, creating a new contract (novation).

## 4. Related work

We discuss here related work in the literature on the representation of contractual dynamics and the representation of (legal) power in general.

Languages of contractual representation such as Contract Language (CL) (Prisacariu and Schneider, 2012), Business Contract Language (BCL) (Governatori and Milosevic, 2006), and FCL (Farmer and Hu, 2016) have been proposed in order to represent contractual clauses dynamics. These languages address contractual dynamics in terms of violation of clauses or, more precisely, violation of the deontic conduct commands *permission, obligation*, and *forbidden* into contractual clauses.

One of the limitations of this approach is that it reduces (or excludes) the relational nature of contractual elements and contracts as a whole. The lack of a representation of the legal relations in the approach adopted by these languages is the result of an ontological choice, i.e., the representation of the legal world is based on monadic operators of *permission (P), obligation (O)*, and *forbidden (F)*. However, the legal world is more complex and cannot be represented only in terms of those operators as clearly explained by Alexy (2009). For instance, suppose that John (j) has as obligation to take an action *a* in face of Carl (c) and also has the obligation not to take the same action *a* in the face of Mary (m). It would be possible to represent these obligations (formally, *O(a)* and *O*(¬*a*)*)*, but it would not be possible to identify for whom action *a* is mandatory and in the face whose, which would lead to inconsistency within the legal system. On the contrary, a relational representation allows the existence of both obligations in the same legal system without creating inconsistencies due to the identification of the subjects bound by the obligation (formally, *OjcA* and *Ojm*¬*A*, where *A* is the action). This representation is relevant in scenarios where, for example, a service provider changes the service contract only for a group of customers (e.g., from a specific date of signing the contract).

Another limitation of these languages is that they only focus on the violations of the commands existing in contractual clauses. Thus, in the preceding example, the contract dynamics would be represented only in terms of which action must bring about in case of violation of clauses; no representation is presented for the case where the contract dynamics occurred by mere application of the clause of power of unilateral contractual change.

In addition, contract formalisms and contract violations based on multi-parties contracts have been investigated in the scope of models and languages, such as Multi-party Contract Model (Xu, 2004) and Contract Specification Language (CSL) (Hvitved, 2011). Multi-party Contract Model is a model to represent multi-party contracts and detects the parties responsible for contract violations. It is composed of three core components, namely, actions, commitments, and a commitment graph. Actions are performed by the contractual parties when playing roles. A commitment is defined as ‘a guarantee by one party toward another party that some action sequence shall be executed completely, and all involved parties fulfill their side of the transaction' (Xu, 2004). A commitment graph is a visual representation of the complex relations among commitments. In this model, contract violations refer to breach to comply with a contractual clause by contractual parties.

Contract Specification Language is a language based on a trace-based model for multiparty contracts, with the possibility to assign the blame to a set of parties. Contractual conformance is defined abstractly as a property on traces. In this language, as in the languages mentioned earlier the contractual dynamics lies down in the obligation violations. An important characteristic of languages such as Contract Language (CL) and Contract Specification Language (CSL) is that they are based on the *ought-to-do* approach rather than the *ought-to-be* approach. As explained by Prakken and Sergot (1996), the first approach is concerned with actions and the second one is concerned with states of affairs. As claimed by the authors, the chosen approach can impact the representation of defeasibility (e.g., contractual clause defeasibility) and violation of primary obligations. In any case, there is no attention to actions that alter the legal positions in the contracts.

Regarding the use of ontologies to model contracts and related aspects, several studies have been proposed in many contexts, including obligation states to obtain contractual information. We point out the Contract Workflow Model (CWM) based on an ontology of contracts called Multi-Tier Contract Ontology (MTCO) (Kabilan, 2005; Kabilan et al., 2005). In this study obligations, obligations states, and performance events are related to each other. This structure is possible to comprehend and trace obligations in contracts, relating them to the expected performance. In that approach, obligations are classified by its state as “active, triggered, pending, and fulfilled” (Kabilan et al., 2005); for each executed contract, a CWM is deduced and compared with the beginning CWM by means of instances of these models.

Other studies along the same line include the Web Ontology for Copyright Contract Management proposed by Garćıa and Gil (2008). This domain ontology focuses on Digital Rights Management (DRM) and interoperability in Internet scenarios. Contract dynamics is based on event patterns, using an *action plus an approach of case roles modeling*, which amounts to the same approach proposed by Linington et al. (2004) and Linington et al. (2012) to verify the violation of contractual clauses.

Although these conduct-norm violation-driven representations are useful to represent a breach of contractual clauses, they are not adequate to represent a type of contractual dynamics, in which there is an alteration of contractual contents *without clause violation*, as in the case discussed in Section 3.3.

Since the previously mentioned languages were not built to represent contractual changes as exemplified by WhatsApp's Terms of Service, what approach would be appropriate to represent these types of contractual changes? We argue that the approach to represent these changes must be able to: 1) represent contractual changes, such as new clauses or alteration of clauses without violation of previously established clauses; 2) represent new clauses or alteration of clauses without creating a new contract (called *contract amendments*); 3) represent relational aspects in order to specify who is the power holder to able of creating or altering contractual clauses in a unilaterally way; and 4) represent not only legal positions of conduct but also legal positions of power and subjection.

There are some languages for contract representation that have these aspects, one of them is Symboleo (Sharifi et al., 2020), which is a formal specification language for contracts, based on UFO-L (Griffo et al., 2019). This language is described in terms of logical axioms on state charts that describe the lifetimes of contracts, obligations, and powers. Although Symboleo represents the legal position *power*, it does not represent all legal positions existing in UFO-L, in particular, the legal position *permission*, which is an important one as we have shown in the representation of Amazon Web Service Agreements (Griffo et al., 2019).

Regarding related studies on *legal power*, the literature is vast on it. Here, we highlight some studies, such as Hohfeld (1913) and Alexy (2009) for they are related to our study, In addition, several types of research in the field of computing has been developed on this topic, among which we highlight the studies on *normative positions* (Sergot, 2013), *powers and permissions* in security systems (Firozabadi and Sergot, 1999), and norm-governed computational societies (Artikis et al., 2009). There are those studies focused on the formalization of *power* or *institutional power*, for instance, (Jones and Sergot, 1996; Gelati et al., 2002; Boella et al., 2004; Gelati et al., 2004; Sartor, 2006a; Boella and van der Torre, 2007).

The concept of legal power (Alexy, 2009), legal competence (Lindahl, 1977; Spaak, 1994, 2009; Lindahl and Reidhav, 2017), or institutional power (Jones and Sergot, 1996) has been widely discussed by researchers in the legal field including (Hohfeld, 1913; Hart, 1976; Halpin, 1996; MacCormick, 1998), as well as researchers in the computational field such as Governatori and Rotolo (2004) and Sartor (2006b), who distinguish different types of power as follows: enabling-power, potestative right, and declarative power; (Boella et al., 2001), by proposing an action-based ontology of legal relations, introducing the idea of *recursion* from *power*, and the creation of obligations from powers. In truth, we agree that legal powers create duties and obligations as proposed by Boella et al. (2001), but we understand that powers create not only obligations but also all types of legal positions, including other legal powers in a secondary level of powers (powers of powers). Our understanding comes from the concept of legal power proposed by Alexy (2009), who understands legal power as a legal position hold by a legal agent and who is able to create new legal positions for the correlated legal position (*subjection*) hold by the correlate legal agent. Thus, person *x* has a power *C* as against person *y* to create a legal position *P* for person *y* (Cxy(Py)). In the case study, new permissions are created by WhatsApp exercising its legal power position, which confirms the need to understand legal powers as generators of permissions, rights, duties, no-rights, etc.

## 5. Final considerations

In this study, we identified the need to address the dynamics of service contracts. While earlier studies on contractual dynamics focused on violations of deontic commands existing in contractual clauses, we focused on contractual dynamics representation based on legal relations, in particular, in legal relations of power and subjections existing in contractual clauses of unilateral changes. In addition, we showed the difference between *amendment* and *novation* and the differences in the representation of these two types of contractual changes. To illustrate our approach, we selected WhatsApp's latest terms of services with amendments.

Notwithstanding the consistent and effective approach used by the related studies for the analysis of contractual clauses and verification of possible breaches, we realized that none of these studies address the representation of the dynamics of contracts beyond those aspects concerning contractual violation of duties and obligations. Our perspective is to model the dynamics of *power* that agents have during the lifecycle of a contract as well as to represent what is created or modified through this exercise of power (e.g., new contractual clauses or exclusion of contractual clauses). From this perspective, the approach used by these previously cited studies is relevant but not sufficient. This is because it is essential to represent not only duties and obligations—as mentioned in the cited related studies, but also permissions, rights, no-rights, liberties, powers, subjections, disabilities, and immunities, all those legal positions that can be held by legal agents in legal relations. Hence, the account proposed in this study identifies an important representation gap in the various contract languages in the literature.

Carefully representing contract dynamics becomes relevant in different situations, one of which is the management of legal contracts in business conglomerates such as Meta, Alphabet, etc. Business conglomerates need to manage legal agreements for different companies in different countries under unlike laws with a huge number of service customers over time. Although we have used here a business-to-consumer scenario, we understand the same motivation applies to business-to-business scenarios, especially in long-running business collaborations.

Thus, this article, despite just scratching the surface of the subject, aims to contribute to legal practice with regard to contract management, computable representation of contractual elements according to contractual content, and automation of reasoning about contractual relations and the existing intrinsic aspects in the various legal roles involved in the contracts.

There are various unexplored issues regarding the dynamics of contractual relations to be addressed in future studies. Some ones that arose during the study were as follows:

1) **Consumer silence as acceptance of contractual changes**: In WTS, the continued use of the service is interpreted as a declaration of will sufficient to give validity to the contractual modification (‘Your continued use of our Services confirms your acceptance of our Terms, as amended'). Consequently, there are only three alternatives as follows: a) consumer does not agree with the modification terms and stops using the service; b) consumer explicitly agrees with the modification terms; or c) consumer continues the use of the service, which is interpreted as an acceptance of the modified terms. Therefore, there is no alternative to continuing to use the service under the previously agreed terms.

2) **What can be changed by a contractual amendment?** The issue of whether a certain contractual change amounts to contract amendment or novation is ultimately a domain-specific one and may be dependent on the particular legal system in place and the nature of the contractual object. Hence, it cannot, in principle, be settled generally at the level of a domain-independent representation scheme as the one explored here. Nevertheless, foundational distinctions–such as the distinction between kinds and phases–along with well-founded representation patterns, *prime the modeler* to address the matter of amendment or novation in a context of the application. Addressing this matter is required to fully capture the particular contractual reality. Constraints to what can count as amendments (or novations) may arise out of legal provisions (such as those discussed here for the European Union Directive 2019/770), case law, or principles in the legal system concerning the essential aspects of a contract (e.g., its ultimate purpose, its objects). They may certainly be the subject of legal controversy, in which case opposing parties may formulate alternate theses concerning the nature of a contractual change. In any case, our representation scheme has the expressive power to capture the intended meaning concerning the contract change, beyond what is revealed at the surface by the language that is employed in the description of contextual changes.

3) **Contractual modification with mutual consensus**: The case of mutual consensus for contractual modifications was not part of this study's scope. However, in future study, we point out an analysis of clauses with balanced power-subjection relations bear by bound parties. This analysis will allow verifying whether theories such as win-win are being applied in the negotiation phase and how this phase can be fine-tuned. This analysis is not a trivial one for the possibility of changing the object of each legal relation of power-subjection.

In conclusion, there are ongoing studies: (1) a systematic study on the theory of contracts, adhesion contracts, and negotiation, correlating studies such as Macaulay (2018) and (Unger, 1977) with studies on adhesion contracts such as Rakoff (1982) and Kessler (1943). This study will provide a broader and deeper legal-theoretical foundation on the topic outlined here; and (2) the ongoing study of an operational modeling pattern of legal relations by means of the Ontoprolog system (Araujo and Lima-Marques, 2017). Ontoprolog was conceived based on UFO's ontological foundations and built on a logical framework of Logic Programming. The application of Ontoprolog using the representational strategy discussed here can leverage the logical treatment of contract representation and allow logical deductions including the dynamic aspects we have discussed here. Another future study is the development of algorithms to support the automated identification of legal position types from contractual clauses.

## Data availability statement

The original contributions presented in the study are included in the article/supplementary material, further inquiries can be directed to the corresponding author.

## Author contributions

JA, GG, and CG conceived of the presented idea and developed the ontological modeling. SM analyzed the types of contractual changes. M-LM and LA developed the operational ontology, verified the analytical methods, and supervised the findings of this work. All authors discussed the results and contributed to the final manuscript.

## Footnotes

^1^'For example, consider that “John” is permitted to “smoke outdoors”. This is a convenient form of expression when deontic constraints apply in an absolute sense, i.e., to the legal system as a whole. Because of this, it leads to simpler formal treatment. Consider, however, that deontic constraints may apply in the scope of some contractual relation but not in another. For example, “John” may be obliged as against his employer not to “drink alcohol while on duty call” but not against other agents. When the same agent is in different contracts, what is forbidden as against one agent may permitted as against another. For example, “Amazon, Inc.” may be permitted to “store data in the U.S.” for some agents but not to others.'

^2^Facebook Companies are currently 78 companies (such as: Crowdtangle, a social analytics service that monitors trending stores, Threadsy, the company that operated a social influencer tool.) Thus, the expression ‘affiliated companies' includes but is not limited to these 78 companies.

^3^The TS and Privacy Policy version lunched on January 4, 2021 does not differentiate Service Customers (Business client account or non-business client account), indicating that the updates apply for all type of Service Customers.

^4^Note, however, that the *Service Customer* has the possibility to change the contractual clauses with the express consent of WhatsApp (see clause Id36 in Table 2 and clause Id29 in Table 4).
